# Argon prevents the development of locomotor sensitization to amphetamine and amphetamine-induced changes in mu opioid receptor in the nucleus accumbens

**DOI:** 10.1186/s13618-014-0021-z

**Published:** 2014-12-29

**Authors:** Hélène N David, Martine Dhilly, Géraldine Poisnel, Mickael Degoulet, Cédric Meckler, Nicolas Vallée, Jean-Éric Blatteau, Jean-Jacques Risso, Marc Lemaire, Danièle Debruyne, Jacques H Abraini

**Affiliations:** Centre de recherche Hôtel-Dieu de Lévis, CSSS Alphonse-Desjardins, Lévis, QC Canada; Département d’Anesthésiologie, Université Laval, Québec, QC Canada; ISTCT UMR 6301, CEA DSV/I2BM, LDM-TEP group, GIP Cyceron, Caen, France; ISTCT UMR 6301, CNRS, Caen, France; ISTCT UMR 6301, Université de Caen Basse-Normandie, Normandie-Université, Caen, France; Université de Caen - Basse Normandie, Normandie-Université, Caen, France; Institut de Recherche Biomédicale des Armées, Toulon, France; Air Liquide, Centre de Recherche Claude-Delorme, Jouy-en-Josas, France

**Keywords:** Amphetamine, Locomotor sensitization, GABA, Benzodiazepine, Argon, Noble gases

## Abstract

Systemic administration of γ-amino-butyric acid type A (GABA-A) and benzodiazepine receptor agonists has been reported to block the development of locomotor sensitization to amphetamine. Here, we investigated whether the non-anesthetic noble gas argon, shown to possess agonistic properties at these receptors, may block the acquisition of amphetamine-induced locomotor sensitization and mu opioid receptor activation in the nucleus accumbens. Rats were pretreated with saline solution or amphetamine (1 mg/kg) from day 1 to day 3 and then exposed, immediately after injection of amphetamine, to medicinal air or argon at 75 vol% (with the remainder being oxygen). After a 3-day period of withdrawal, rats were challenged with amphetamine on day 7. Rats pretreated with amphetamine and argon had lower locomotor activity (*U* = 5, *P* < 0.005) and mu opioid receptor activity in the nucleus accumbens (*U* = 0, *P* < 0.001) than rats pretreated with amphetamine and air. In contrast, argon had effect on locomotor and mu receptor activity neither in rats pretreated with saline and challenged with amphetamine (acute amphetamine) nor in rats pretreated and challenged with saline solution (controls). These results indicate that argon inhibits the development of both locomotor sensitization and mu opioid receptor activation induced by repeated administration of amphetamine.

## Introduction

Over the past 10 years, a series of in vitro and in vivo studies has demonstrated the organoprotective and therapeutic potential of the inert gases xenon, nitrous oxide, and argon [1-13]. Particularly, in line with their antagonistic action at the N-methyl-D-aspartate (NMDA) glutamate receptor and nicotinic acetylcholine (nACh) receptor [14-19], xenon and nitrous oxide at subanesthetic doses have been shown to block the development of locomotor sensitization to amphetamine [20], which is characterized by an enhanced locomotor response to an amphetamine challenge in rats pretreated with repeated amphetamine. So far, in contrast with xenon and nitrous oxide, the non-anesthetic gas argon is thought to act mainly through activation of the γ-amino-butyric acid (GABA) type A and benzodiazepine receptors [21]. Although GABA-A and benzodiazepine receptor agonists have been shown to block the acquisition of locomotor sensitization to amphetamine and amphetamine-derived drugs [22,23], whether argon may also inhibit the development of locomotor sensitization to amphetamine still remains unknown.

Therefore, in the present study, we investigated the effect of argon on the development of amphetamine-induced locomotor sensitization and mu opioid receptor activation the nucleus accumbens, whose elevated activity has been shown to be critically involved in the development of neurobehavioral sensitization to amphetamine [24-27].

## Materials and methods

### Animals

All animal-use procedures were in accordance with the Declaration of Helsinki and within the framework of the French legislation for the use of animals in biomedical experimentation, and were approved by a research ethic committee. Male adult Sprague–Dawley rats (Janvier, Le Genest Saint-Isle, France) weighing 250 to 300 g were used. Rats were housed socially at 21.5°C in groups of 3 to 4 in perspex home cages with free access to food and water. Light was maintained on a reverse light–dark cycle, with lights on from 8 PM to 8 AM.

### Sensitization to amphetamine

**Figure 1:**
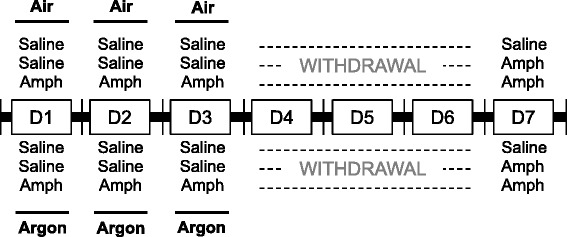
**Experimental protocol and procedures.** Rats (n = 8 per group) were treated daily with either saline solution (1 mL/kg intraperitoneally; IP) or amphetamine (1 mg/mL/kg, IP) from day 1 to day 3. Immediately after injection of saline solution or amphetamine, rats were treated for 3 h with “medicinal” air (composed of 75 vol% nitrogen + 25 vol% oxygen) or argon at 75 vol% (with the remainder being oxygen). Then, after 3 days of withdrawal, behavioral investigations were performed on day 7. Immediately after behavioral testing, the rats’ brain were removed and used to assess the constitutive activity of the mu opioid receptor in the nucleus accumbens.

Rats (*n* = 8 per group) were treated daily with either amphetamine (1 mg/mL/kg, intraperitoneally; IP) or saline solution (1 mL/kg, IP) from day 1 to day 3. Immediately after injection of amphetamine or saline solution, rats were treated for 3 h with “medicinal” air (composed of 75 vol% nitrogen + 25 vol% oxygen) or with argon at 75 vol% (with the remainder being oxygen). Then, after 3 days of withdrawal, behavioral and neurochemical investigations were performed as detailed below (Figure 1). Both gas mixtures were administered at a flow rate of 5 L/min in a closed chamber of 100 L volume (65 × 45 × 35 cm), a condition that allows maintaining carbon dioxide less than 0.03 vol% and humidity about 65-70% inside the chamber with the use of soda lime and silica gel, respectively.

### Behavioral investigations

On day 7, rats were habituated to the activity boxes for 1 h before being challenged with saline solution (1 mL/kg, IP) or amphetamine (1 mg/mL/kg, IP), and then were recorded for locomotor activity for 1 h 30 min as detailed previously [21]. Locomotor activity was quantified using a bank of 4 individual activity cages measuring 30 × 20 × 20 cm, equipped with horizontal infrared beams, located 3 cm above the floor across the long axis of the cage (Imetronic, Pessac, France). Beam interruptions were detected through an electrical interface and recorded over 10-min intervals. All experiments were performed during the animals’ dark cycle with the activity boxes kept dark.

### Neurochemical assays

Immediately after amphetamine challenge on day 7, rats were killed and their brain was carefully removed from their skull, frozen with isopentane and then stored at −20°C. For each animal, two coronal sections of 20 μm thickness including the nucleus accumbens (anteriority: +1 mm from the bregma) were cryostat cut, applied to glass slides with a very low non-specific binding capacity (Superfrost Plus, Menzel-Glaser GmbH, Braunscheig, Germany) and stored at -20°C until required for the binding assays.

Saturation binding was performed on rat brain sections as detailed previously [28]. Brain sections were preincubated twice for 5 min at 4°C in 50 mM Tris–HCl buffer solution [(hydroxyl-methyl)aminomethane] containing 100 mM NaCl, 1 g/L bovine serum albumin (BSA), and 20 mg/L bacitracin, adjusted to pH 7.4, in order to dissociate and eliminate potential endogenous ligands. Then, brain sections were incubated for 45 min at 4°C using 800 μL of buffer solution containing increasing concentrations (0.312, 0.625, 1.25, 2.5, 5 nM) of [^3^H]DAMGO [(D-ala^2^,N-methyl-phe^4^,glycol^5^)(tyrosyl-3,5-^3^H)enkephalin, 1 Ci/L, specific radioactivity 66 Ci/mmol]. The amount of non-specific labelling was assessed using adjacent brain sections in the presence of an excess of naloxone at 10 μM. After incubation, brain sections were quickly washed (30 s) with Tris–HCl buffer containing BSA (x1) and then with Tris–HCl buffer alone (x3) at 4°C in order to eliminate unbound ligand. A final wash was performed at 4°C with distilled water to remove excess of buffer salts. Then, brain sections were dried over night at room temperature and stored until counting. Before being used for image acquisition and data analysis, slides containing brain sections were exposed under tritium-sensitive phosphor screens in the dark for 10 days at −20°C. Images were then captured with a computer-controlled Cyclone phosphorimaging scanner using the OptiQuant acquisition and analysis software (Packard Instrument Company, Meriden, CT, USA). Optical densities expressed as digital light units per mm^2^ over [^3^H] standard spot were measured. Specific binding was determined by subtracting non-specific binding from total binding. Saturation binding data were fitted according to a one site binding (hyperbola) model using Graph Pad prism (Graph Pad Prism 4.02; Graph Pad Software, La Jolla, CA, USA). The ratio of changes in percentage from basal values of the maximal number of binding sites (Bmax in fmol/mm^3^) to the dissociation constant (Kd in nM) was calculated by the computer to assess the level of constitutive activity of the mu opioid receptor in the nucleus accumbens in the presence of amphetamine and/or argon.

### Drugs, chemical, and gases

Amphetamine (d-amphetamine hemisulfate salt, ref. A5880), BSA (bovine serum albumin ref. 2153), and naloxone (Naloxone hydrochloride dihydrate, ref. N7758) were purchased from Sigma-Aldrich (Illkirch, France). Bacitracin was purchased from MP biomedicals (Santa Ana, CA, USA), and [^3^H]DAMGO (66 Ci/mmol) from Amersham Biosciences (Buckinghamshire, UK). Oxygen, nitrogen and argon of medicinal grade were purchased from Air Liquide (Paris, France). Gas mixtures composed of 75 vol% nitrogen + 25 vol% oxygen or 75 vol% argon + 25 vol% oxygen were obtained using calibrated flowmeters and gas analysers.

### Data presentation and statistical analysis

Data were expressed as the median and quartiles values, and analyzed using non-parametric statistical methods. Between-group comparisons were performed using the Kruskall-Wallis analysis of variance; following a significant H value, *post hoc* analysis was performed using the Mann–Whitney *U-*test. Statistical significance was set at *P* ≤ 0.05.

## Results

### Amphetamine-induced changes in locomotor activity and Mu receptor activity

**Figure 2:**
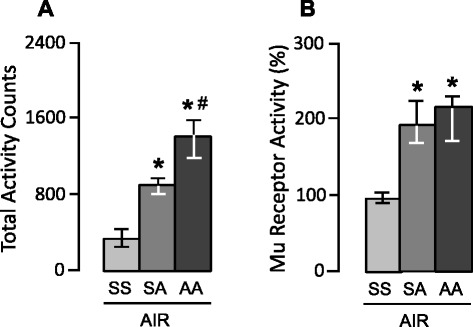
**Effects of amphetamine on locomotor activity and mu receptor activity in the nucleus accumbens. (A)** When challenged with amphetamine, rats pretreated with repeated administration of saline solution (SA) or amphetamine (AA) had higher locomotor responses than rats pretreated and challenged with saline solution (SS). Locomotor activity is expressed in arbitrary units. **(B)** As assessed immediately after being challenged with amphetamine, rats pretreated with repeated injection of saline solution (SA) or amphetamine (AA) had higher mu receptor activity in the nucleus accumbens than rats pretreated and challenged with saline solution (SS). The ratio of the dissociation constant (Kd) to the maximal number of binding sites (Bmax) was calculated to assess the activity of mu receptors in the nucleus accumbens; mu receptor activity in controls rats pretreated and challenged with amphetamine was taken as a 100 % value. SS: pretreatment with saline + challenge with saline; SA: pretreatment with saline + challenge with amphetamine; AA: pretreatment with amphetamine + challenge with amphetamine. * *P* < 0.001 *vs* SS + Air; ^**#**^ *P* < 0.001 *vs* SA + Air.

The effects of amphetamine on locomotor activity and mu receptor activity are illustrated in Figure 2. All rats were pretreated with either saline solution or amphetamine and then exposed immediately to medicinal air used as a control gas treatment. When challenged with amphetamine, rats pretreated with repeated administration of saline solution or amphetamine had higher scores of locomotor activity than control rats pretreated and challenged with saline solution (*U* = 1, *P* = 0.001, Figure 2B). Further comparison between rats challenged with amphetamine showed that rats pretreated with repeated administration of amphetamine had higher scores of locomotor activity than rats pretreated with saline (*U* = 0, *P* < 0.001, Figure 2A), indicating that locomotor sensitization to amphetamine had occurred.

As assessed immediately after the amphetamine challenge, rats pretreated with repeated administration of saline solution or amphetamine had increased mu receptor activity as estimated by the ratio of Bmax to Kd compared to control rats pretreated and challenged with saline solution (*U* = 4, *P* = 0.005; U = 0, *P* < 0.001; Figure 2B). However, in contrast with what seen for locomotor activity, further comparison between rats challenged with amphetamine revealed no significant difference in mu receptor activity between rats pretreated with repeated administration of amphetamine and those pretreated with repeated administration of saline solution and medicinal air (*U* = 32, *n.s.*, Figure 2B).

### Effects of argon on amphetamine-induced changes

**Figure 3:**
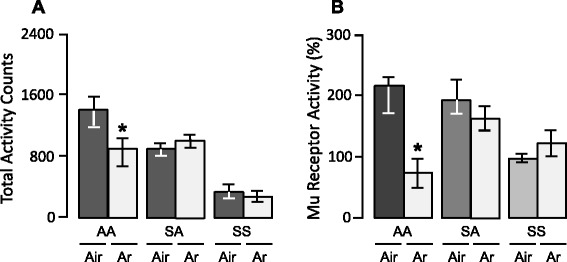
**Effects of argon on amphetamine-induced changes in locomotor activity and mu receptor activity in the nucleus accumbens. (A)** When challenged with amphetamine, rats pretreated with amphetamine and argon had lower locomotor activity than rats pretreated with amphetamine and air (AA); in contrast, no significant difference in locomotor activity was found between rats pretreated with saline and argon and those pretreated with saline and air when challenged with amphetamine (SA) or saline (SS). This indicates that argon blocked locomotor sensitization to amphetamine, but had effect neither on locomotor activity induced by acute amphetamine nor on basal locomotor activity. Locomotor activity is expressed in arbitrary units. **(B)** As assessed immediately after being challenged with amphetamine, rats pretreated with amphetamine and argon had reduced mu receptor activity compared to rats pretreated with amphetamine and air (AA); in contrast, no significant difference in mu receptor activity was found between rats pretreated with saline and argon and those pretreated with saline and air when challenged with amphetamine (SA) or saline (SS). This indicates that argon blocked the increase in mu receptor activity induced by repeated amphetamine, but had effect neither on the increase in mu receptor activity induced by acute amphetamine nor on basal mu receptor activity. The ratio of the dissociation constant (Kd) to the maximal number of binding sites (Bmax) was calculated to assess the activity of mu receptors in the nucleus accumbens; mu receptor activity in controls rats pretreated and challenged with amphetamine was taken as a 100 % value. SS: pretreatment with saline + challenge with saline; SA: pretreatment with saline + challenge with saline; AA: pretreatment with amphetamine + challenge with amphetamine. * *P* < 0.001 vs AA + Air.

The effects of argon on locomotor sensitization and changes in mu receptor activity induced by repeated administration of amphetamine are illustrated in Figure 2. Exposure to argon, immediately after administration of amphetamine, blocked the development of locomotor sensitization to amphetamine. Indeed, when challenged with amphetamine, rats pretreated with amphetamine and argon had lower locomotor activity than control rats pretreated with amphetamine and air (*U* = 5, *P* < 0.005; Figure 3A). In contrast with its inhibitory effect on the development of locomotor sensitization to amphetamine, argon had significant effect neither on the locomotor-activating action of acute amphetamine nor on basal locomotor activity (Figure 3A). Indeed, when challenged with amphetamine, rats pretreated with saline solution and argon had locomotor activity that was not different from that displayed by rats pretreated with saline solution and air (*U* = 20, *n.s.*). Likewise, when challenged with saline solution, rats pretreated with saline solution and argon had locomotor activity that was not different from that displayed by rats pretreated with saline solution and air (*U* = 17.5, *n.s.*).

Exposure to argon immediately after amphetamine administration blocked the increase in mu receptor activity induced by repeated administration of amphetamine, so that, as assessed immediately after the amphetamine challenge, rats pretreated with amphetamine and argon had reduced mu receptor activity compared to rats pretreated with amphetamine and air (*U* = 0, *P* = 0.001, Figure 3B). In contrast, argon had no significant effect on mu receptor activity in rats pretreated with saline and air and challenged with amphetamine (acute amphetamine; *U* = 32, *n.s*., Figure 3B), or in control rats pretreated and challenged with saline (*U* = 19, *n.s.*, Figure 3B).

## Discussion

In the present study, we showed that argon inhibited the development of locomotor sensitization to amphetamine, but had effect neither on the locomotor activating properties of acute amphetamine nor on basal locomotor activity. In addition, we found that argon further blocked the increase in mu receptor activity induced by repeated administration of amphetamine, but had effect neither on the increase in mu receptor activity induced by acute amphetamine nor on basal mu receptor activity in the nucleus accumbens. Taken together, these results show that argon “specifically” inhibits both locomotor sensitization and the increase in mu receptor activity induced by repeated amphetamine administration.

The mu opioid neurotransmission is an integral part of the motive circuit and as such it is well recognized to be fully involved in the mechanisms of action and the behavioral effects of drugs that belong to the amphetamine family [27]. Thus, rats subjected to repeated administration of amphetamine have been demonstrated to exhibit enhanced responsiveness and elevated constitutive activity of mu opioid receptors in the nucleus accumbens [29], and knock-out mice lacking the mu receptor have been shown to be insensitive to behavioral sensitization induced by amphetamine-derived drugs [26]. Interestingly, other investigations have shown that the GABA-A and mu receptors are closely linked. In that way, previous studies have demonstrated that blocking the mu receptor by specific antagonists allows inhibiting behavioral responses elicited by focal injection of GABA-A receptor agonists in the nucleus accumbens [30]. Also, mu-opioid receptor knockout mice have been reported to show increased binding for [^3^H]GABA-A agonists in the cortex and hippocampus compared to wild-type mice [31]. Therefore, whether argon inhibits the enhanced constitutive activity of mu receptors induced by repeated administration of amphetamine directly through competitive or non-competitive antagonism of the mu opioid receptor or indirectly through its agonistic action at the GABA-A receptor [21], still remains to be elucidated, particularly if one considers that the administration of prototypical GABA-A and benzodiazepine receptor agonists has been reported to block the acquisition of locomotor sensitization to amphetamine [22,23].

Inert gases are well-known to act at multiple targets. For instance, xenon and nitrous oxide have antagonistic properties at the NMDA glutamate and nACh acetylcholine receptors and, on the contrary, possess agonistic action at the two-pore domain TREK-1 potassium channel [15,16,32]. The present study suggests that argon could act as a mu receptor antagonist in addition of its agonistic properties at the GABA-A receptor [21]. These latter effects could both participate to the inhibiting action of argon at blocking locomotor sensitization to amphetamine [22,23,26,33]. Further in vitro experiments should be performed to investigate the way by which argon modulates the mu opioid receptor, directly or indirectly through activation of the GABA-A receptor. Whatever, our results clearly demonstrate that argon down regulates the activity of the mu opioid receptor and that it may have beneficial effect on the expression of behavioral sensitization to amphetamine, a condition necessary for actually evaluating the potential of argon as a possible therapeutic agent in the treatment of drug addiction.

If argon would act as a direct inhibitor of the mu opioid receptor, its therapeutic potential could be of interest in other, mainly psychiatric, diseases such as depression, stress-induced disorders, attentional and hyperactivity disorders, and impulse control disorders.
